# Supplementary material for: Developing an implementation intervention, and identifying strategies for integrating health innovations in routine practice: A case study of the implementation of an insulin patient decision aid
Source: PLoS One. 2024 Nov 15;19(11):e0310654. doi: 10.1371/journal.pone.0310654 (PMC11567623; doi:10.1371/journal.pone.0310654)
Supplement: S1 Table — (DOCX) [file pone.0310654.s001.docx]

**S1 Table**

**Voting form**

**Below are barriers that may hamper the implementation of the insulin PDA in your clinic.**

**Instructions:**

1. Please read each statement carefully and think which of the barrier would hamper

the implementation of the insulin PDA in your clinic.

2. If there are any statements that you are unclear of, please feel free to ask any of the

study team member.

3. Please indicate your answer by providing a tick in the ‘tick’ column

4. You may tick as many as you desire

|  | **I think HCPs will not use the PDA in the clinic because...** | **[✔]** |
| --- | --- | --- |
| 1 | they don't know where to get the PDA |  |
| 2 | it is difficult to implement the PDA in the clinic because there is a lack of funding to print the PDA booklets |  |
| 3 | they will not be able to see the same patient to follow up on the PDA |  |
| 4 | they already have their own way of doing things |  |
| 5 | there are many other health programmes in the clinic |  |
| 6 | there is no clear directive / circular from the top management to use the PDA |  |
| 7 | they are too busy as there are too many patients |  |
| 8 | there is no one championing the use of PDA in the clinic |  |
| 9 | senior persons in the clinic do not motivate them to use it |  |
| 10 | they are not the person-in-charge to use of the PDA in the clinic |  |
| 11 | using PDA is not part of their job scope |  |
| 12 | they do not want the patient to ask them more questions after using it |  |
| 13 | they are not aware of the insulin PDA |  |
| 14 | using the insulin PDA is extra work for them |  |
| 15 | they are not familiar with the insulin PDA booklet (e.g: relevance, content, usage) |  |
| 16 | they want to finish their work quickly |  |
| 17 | they tend to make decisions for their patients instead of practising shared decision making |  |
| 18 | they believe that insulin PDA is not useful for improving patient care and outcomes |  |
| 19 | they think that it is not credible (reliable and trustworthy) |  |
| 20 | they are not motivated to try new innovations |  |
| 21 | they will forget to use it |  |
| 22 | they heard negative things about the insulin PDA |  |
| 23 | their colleagues influence them not to use the PDA |  |
| 24 | they are concerned that the PDA may delay patients' treatment decision |  |
| 25 | they are concerned that the PDA may replace their consultation |  |
| 26 | they are concerned about giving patient the options of "alternative treatment" or "do nothing" |  |
|  | **I think patients will not use the PDA because...** |  |
| 27 | they cannot read or understand the PDA |  |
| 28 | they are not willing to pay for the PDA |  |
| 29 | it has too many pages |  |
| 30 | they are not confident to use the PDA by themselves |  |
| 31 | they are not motivated to use it |  |
| 32 | it is too wordy |  |
| 33 | they rely on doctors to make health decisions |  |
| 34 | they do not like to read |  |
| 35 | they feel that the PDA is a tool to persuade them to start insulin |  |
| 36 | it is confusing |  |
| 37 | their poor vision makes it difficult to read the PDA |  |
| 38 | they are too busy |  |
| 39 | they do not trust the doctor |  |
| 40 | it makes patients feel anxious |  |
| 41 | it is not visually attractive |  |
| 42 | it is not informative enough |  |
| 43 | they have to make extra trips to the clinic for PDA follow up |  |
| 44 | they feel that the PDA is not useful |  |
|  |  |  |
|  | **Patients have difficulty to use the PDA with HCPs because…** |  |
| 45 | of language barrier |  |
| 46 | HCPs are not clear when giving information to them |  |
| 47 | HCPs talk too fast |  |
| 48 | HCPs use difficult words when talking to patients |  |
|  |  |  |
|  | **It is hard to use the PDA in the clinic because…** |  |
| 49 | there is not enough rooms in the clinic for PDA discussions |  |
|  |  |  |
