# Supplementary material for: Developing an implementation intervention, and identifying strategies for integrating health innovations in routine practice: A case study of the implementation of an insulin patient decision aid
Source: PLoS One. 2024 Nov 15;19(11):e0310654. doi: 10.1371/journal.pone.0310654 (PMC11567623; doi:10.1371/journal.pone.0310654)
Supplement: S2 Table — (DOCX) [file pone.0310654.s002.docx]

**S2 Table**

**Strategies proposed by Phase 1 participants to facilitate the insulin PDA implementation**

| **Strategy** | **Description** | **Representative quote** |
| --- | --- | --- |
| 1. HCPs to inform the purpose and the benefits of the insulin PDA to patients | Patients may not be keen to use the insulin PDA when they do not know what it entails and how it can benefit them. HCPs should introduce and explain to patients the purpose and advantages of using the insulin PDA. | *“You need to explain to the patient because if you just put it (the PDA) there, they won’t know what is inside. Like I come for check-up, a nurse recommend ‘We are having a new book. Good for your health so maybe you can change’.” – Community clinic A_Patient 3* |
| 1. HCPs to go through the insulin PDA with patients | HCPs should spend time to explain the insulin PDA and go through it with patients to clarify their doubts. | *“No matter what we give to the patients, we need to make ourselves available to answer their queries. Maybe there are some information that they are not sure.” – UMMC_Doctor 3* |
| 1. HCPs to press on insulin PDA use among patients | Doctors need to press on the importance of using the insulin PDA with patients so they take it seriously. | *“I think if the doctor tells me ‘You seriously read about this and get back to me. Think carefully, this is going to help you.’. I think I will use it. The doctor plays an important role here.” – UMMC_Patient 1* |
| 1. To get HCPs to introduce the insulin PDA to patients | Patients would rather trust to accept the insulin PDA from HCPs, especially doctors, than people outside of the medical profession. | *“For my generation, we always look up to doctors. Whatever doctor says means it’s correct. I would accept it. If I received it from a doctor, I would pay attention to it.” –UMMC_Patient 9* |
| 1. Involve patients’ family members or caretakers | Many participants suggested engaging with family members or caretakers when using the insulin PDA during consultations. Based on their experience, it’s helpful due to communication barriers and dealing with patients who cannot read or understand the PDA. | *“I can’t speak Tamil so I will have to rely on the patient’s carer. The easiest way is to get carers involved. Sometime carers come with the patient during consultation so I engaged them with the booklet.” – UMMC_Doctor 8*  *“If there is a relative with them is much better. Older persons may have vision problems. They need to have someone with them. Whenever I teach, I prefer their family members to be there as well because the patient needs support. Their family members must be able to understand the information, so when two people read and of the same opinion then it is easier to make decision.” – UMMC_Staff nurse 1* |
| 1. Inform HCPs on the advantages of the insulin PDA use | Inform HCPs on the advantages of using the insulin PDA in order to motivate them to use. | *“Conduct talks to inform the staff of the importance of this booklet. Explain about it and once the staff understand, they will start using it.” – UMMC_Diabetes educator 3* |
| 1. Refer patients to HCPs who can speak the same language as the patients | To overcome language barrier issues, patients should be referred to HCPs who speak the same language as them. | *“The diabetes educator can only speak in Malay and English so they might have to send the patient to another diabetes educator who can speak the patient’s language.” – UMMC_Doctor 13* |
| 1. Get HCPs to communicate to patient about the insulin PDA in a non-threatening manner | HCPs should deliver the PDA and approach the subject of insulin use in a non-threatening way with patients. HCPs should promote the PDA in a positive light to encourage the patients to use it. They should also take time to explain how to use the insulin PDA to patients in a clear, direct, and layman's language. | *“You should let the patient know that using insulin is not the end game. Have a more pleasant way of approaching the subject so that the patient doesn’t feel threatened, in the sense that insulin is the last resort. Give the PDA with the impression that is not end game but that the patient can even get better. // Speak positive things about it. Motivate the patient.” – UMMC primary care clinic_Patient 9* |
| 1. Reminders | When HCPs are reminded repeatedly, this will prompt them to adopt the insulin PDA. | *“I think it is kept being reminded that there is such a thing.” – UMMC_Healthcare policymaker 1* |
| 1. Advertise the insulin PDA by putting up posters, banner, or notice or advertise on TV in the clinic | To create awareness on the insulin PDA, strategies include the use of mass media to advertise the insulin PDA through posters, on TVs, and through campaigns were suggested. | *“Put posters in the clinic so that carers and patient can see. Then, patient also can remind doctors about the book.” - UMMC_Doctor 13* |
| 1. Conduct campaign | Patients also proposed conducting campaigns to create awareness on the availability of the insulin PDA. | *“Conduct campaign to inform that there is a new book.” – FGD 15_Community clinic D_ Patient 1* |
| 1. Conduct SDM and insulin PDA training | Training sessions to equip HCPs with the necessary knowledge and skills were proposed. For example, understanding how to use the insulin PDA and explaining the concept of SDM. | *“There would be a need for training for the doctors to understand what is SDM. The PDA can be alien to the doctors. They have never seen this ‘What am I supposed to do with this?’. They need to know what to do with it.” – Community clinic E_Healthcare policymaker* |
| 1. Conduct educational group sessions for patients | Administering the insulin PDA in a patient group session was proposed in view of time constraints during doctor consultation times. Patients who forget to read the PDA at home can also use the session as an opportunity to raise concerns and questions that can be clarified immediately. | *“Conduct seminar. Means on a pre-selected date the patients will come. I would definitely come [laugh]. Then save the doctors’ time.” – FGD 10_UMMC primary care clinic_ Patient 2*  *“There are some who probably take back the book and forget to bring. But do it in a group setting of insulin priming by the staff, that is also quite good because of the time constraint and all patient don’t want to take back the book. Patient may say ‘I got no time (to read the PDA)’ so that means during that setting I must be able to see.” - Community clinic D_Doctor 1* |
| 1. To involve diabetes educators or nurses to use insulin PDA with patients | HCPs such as diabetes educators or nurses can be trained to use the insulin PDA with patients. Due to the limited consultation times, doctor can introduce the PDA to patients and then refer them to nurses or diabetes educators to further explain the PDA in detail. | *“Your doctor can assign the patient to a diabetic expert so you reduce the time.”- UMMC_ Patient 11* |
| 1. Systematic documentation | To ensure patients who had already received the insulin PDA would have a follow-up on their PDA usage, many raised the need for documentation. | *“Usually we see other doctor’s patients. So we are not sure how much of the information the previous doctor had informed the patient. So we don’t want to repeat again the same thing again. So I think if we want to implement, then we need to make note like ‘Book given’, ‘KIV to start insulin’ or ‘advice given’. We have to make some notes so that the next doctor knows and can follow up on the PDA.” – UMMC_Doctor 1* |
| 1. Monitor and provide feedback | Many HCPs noted that providing feedback on the number of PDAs that have been given may influence doctors to distribute more PDAs to patients. | *“If there is some sort of feedback mechanism to actually check on how many books have been given then I think we will give to more patients.” – UMMC_Doctor 7* |
| 1. Have a champion | Have a champion who could convince other staff to use the insulin PDA. | *“Somebody influential to start first. Then that person can tell and support their subordinate to try it out.”* *– UMMC_Diabetes educator 1* |
| 1. Place the insulin PDA booklets in doctors’ consultation rooms | The insulin PDA should be placed in the doctors’ consultation rooms for accessibility and as a reminder to use it. | *“Put the books in the consultation room because if you have to get out of your room to look for nurse, this will take time.” – IDI 7_UMMC primary care clinic_Healthcare policymaker*  *“Need to place the insulin PDA on the table [laughs]. The thing needs to be seen all the time (to remind them to use).” - Community clinic B_Diabetes educator* |
| 1. Get sponsorship from pharmaceutical company or diabetes associations | Some participants suggested obtaining sponsorship from private companies, diabetes-related associations, or pharmaceutical companies to financially support and maintain the insulin PDA implementation. | *“To maintain it, you need funding. Maybe we can ask diabetes associations or a company that has an educational grant. For example, Pharma A for their educational research. I think we might need to look at that as well.” - UMMC_Healthcare policymaker 1* |
| 1. Issue directive to implement the insulin PDA | Issuing a directive was noted to be the most effective way to get HCPs to implement the insulin PDA, as they would be compelled to do it. | *“Directive (laugh). That’s the only way we can make it happen. If you make it compulsory then they will try and do it. But once they see the benefits, they will start using it without asking why I need to do this.” – Community clinic C_Healthcare policymaker* |
| 1. Provide incentive or reward | Provision of incentives or rewards to doctors who adopt the insulin PDA would make them feel acknowledged and appreciated. This would motivate them to use the insulin PDA. | *“You can give incentive or acknowledgement to doctors who use the PDA. So they will feel excited about using it and at the same time feeling appreciated.” – UMMC_Diabetes educator 4* |
| 1. Incorporate PDA use as part of key performance index, standard operating procedures or in the diabetes management guideline | Incorporating the insulin PDA use in staff key performance index, clinic’s standard operating procedures, or guidelines for diabetes management can influence HCPs to implement the insulin PDA. | *“You can make it like it is a compulsory thing to do. For example, we must refer diabetic patients to one-stop diabetic centre for the eye and foot check. So this PDA can be a part of that. Put in the standard operating procedure then the doctor will follow.” –UMMC_Doctor 1* |
| 1. Create the insulin PDA implementation flow | Creating an implementation pathway was suggested to help HCPs know the implementation processes as well as their respective tasks in the insulin PDA implementation. | *“Create a work flow so everyone like pharmacist, diabetes educator or doctors would know their roles. When we see the insulin PDA we will know what to do”* – *Community clinic B_Diabetes educator* |
| 1. To have a person in charge for the insulin PDA implementation | Having a person in-charge of the insulin PDA implementation was proposed so others can seek help regarding the implementation. As the individuals who are responsible for the insulin PDA implementation would be given the allocated time to use the insulin PDA with patients, this can help to address the issue of time constraint in the clinic. | *“Because of the lack of time and manpower here, it is very time consuming to actually explain to patients. So is better that there is a designated person to actually counsel, explain to them but then this involves manpower.” - Community clinic C_Pharmacist* |
| 1. To lend the insulin PDA to patients | To reduce the insulin PDA printing cost, some participants suggested a lending system whereby patients could borrow the PDA. | *“Lend the PDA to patient. After reading, they may not read again. This helps to save money. Is not cheap.”* – *IDI 31_Community clinic A_ patient 1* |
| 1. HCP to focus only on important topics when using the insulin PDA with patients | To address the time constraints in using the PDA during the limited consultation time, many participants suggested that doctors could focus only on important topics or issues rather than going through the entire PDA with the patient. | *“There are a lot patients waiting for us so usually we just run through the salient points. We reply to what the patient ask us. Then patient can take the book and read at home. If any problem, we can discuss again.” – UMMC_Doctor 11* |
| 1. To let patients read the insulin PDA at their own time and discuss in the next visit | Many also felt that in view of the time constraints in the clinic, doctors would not be able to use the PDA in detail with patients. Rather, patients should be given the PDA to read on their own as they may need time to go through the information, discuss with their family members and friends. Then, the insulin PDA can be discuss with patients in their next visit. | *“I think this is not something they go through page by page with the patient. It is going to be something like ‘Look, there’s something for you to read. Why don’t you read and the next time you come back, we will discuss it’. So, if they have certain questions, they will point out and it gives you a more directed discussion. If I were to use it, I am not going to go through it page by page. I will probably be looking at my watch and say ‘Oh, no this was taking too long’.” – UMMC_Healthcare policymaker 1* |
| 1. To get patients to use the insulin PDA prior to consultation | Patients can be given the insulin PDA while they are waiting to enter the consultation room as this can enable effective use of patient’s waiting time as well as prepare patients for insulin PDA discussions prior to seeing the doctor. | *“Probably you catch them at the waiting bay and the nurses can give to patient. Then by the time they go into the consultation room, they would have read clearly and understood because we’re chasing for time.” – Community clinic E_Healthcare policymaker* |
| 1. To give an earlier appointment to patients for the insulin PDA follow-up | A patient’s next appointment that is far away might render them to forget about the insulin PDA hence it was suggested that an appointment between 2 weeks to one month would be needed. | *“I think if you are going to give this PDA to patient then the appointment must be earlier instead of the usual 4-5 months because we want to assess whether they are ready or not to be on insulin. So give shorter appointment, maybe 2-4 weeks. // But if you give 3-4 months appointment, certainly they will forget.” – Community clinic B_Healthcare policymaker* |
| 1. Juxtapose PDA in preferred language with patient’s PDA in their preferred language to help with translation | To overcome language barrier, a few doctors suggested juxtaposing PDA with patient’s PDA in their preferred language to overcome language barriers. This strategy had been adopted by some doctors and was felt to be effective. | *“If I have a Chinese patient, he/she will point out to me this word (in the PDA in their preferred language), then I can translate (using my own PDA) then they will know I’m talking about hypoglyacemia. I don’t know how to say hypoglycaemia in Chinese right, so then I just go to the section on hypoglycaemia in the PDA and then show. So it had been very useful for me in this sense.”- UMMC_Doctor 3* |
| 1. To identify patients who are eligible to use the insulin PDA | To select patients who would be willing to participate in SDM, are able to read and understand, and want to use the insulin PDA. | *“You might need to choose your patient correctly. Patients who are going to benefit, knowledgeable, can read. Those who are quite proactive in the sense that they want to do share decision making. Otherwise I don’t think some will even bother.” – Community clinic B_Healthcare policymaker* |
