# Supplementary material for: Developing an implementation intervention, and identifying strategies for integrating health innovations in routine practice: A case study of the implementation of an insulin patient decision aid
Source: PLoS One. 2024 Nov 15;19(11):e0310654. doi: 10.1371/journal.pone.0310654 (PMC11567623; doi:10.1371/journal.pone.0310654)
Supplement: S3 Table — (DOCX) [file pone.0310654.s003.docx]

**S3 Table**

**Justification for strategies selected based on evidence in the literature and the clinic context**

| **Strategy: Mandate change** | This strategy was selected to address the barrier ‘Not having a clear directive from the top management’ to use the insulin PDA. This strategy had been defined as “*have leadership declare the priority of the innovation and their determination to have it implemented”* (Powell et al., 2015, p. 9). This strategy was selected because it has been shown that when organisational leader showed clinicians that SDM was an important organisational priority, it led clinicians to believe that SDM was part of the organisation’s work practice and not something that was imposed on them (Hsu, Liss, Westbrook, & Arterburn, 2013; Joseph-Williams et al., 2017; Lloyd, Joseph-Williams, Edwards, Rix & Elwyn, 2013) **(evidence).** Based on the clinic context, the Head of Department or the clinic coordinator organise monthly unit meetings, where all clinic staff (doctors, diabetes educators, staff nurses, appointment clerks) gathers to discuss matters related to the clinic, Hence, this provides opportunity to implement this strategy in a unit meeting **(clinic context).** Participants from Phase 1 also noted that directive from higher authority was an effective way to implement the insulin PDA. Furthermore, this strategy was perceived to be more feasible compared to other strategies such as incorporating the insulin PDA use as part of key performance index or standard operating procedure. This is because the latter strategies would require approvals from hospital authorities and bureaucratic paperwork that are time consuming **(clinic context).** Declaration from the clinic authority may influence the clinic staff to see that the insulin PDA implementation is a clinic’s priority and will influence them to be involved in the implementation. The expected mechanism of action for this strategy is social influence **(mechanism).** |
| --- | --- |
| **Strategy: Training workshop** | Conducting training workshop was selected to address the barrier ‘HCPs tend to make decisions for their patients’. The expected mechanism of action for this strategy is knowledge, awareness, skills, and beliefs about consequences **(mechanism).**  In many PDA implementation studies, training workshops was found to helped HCPs understand how SDM differed from their current ways of working and shifted their thinking from provision of information to provision of support to patients and to consider patient values ( Joseph-Williams et al., 2017; Stacey et al., 2015; Wirrmann & Askahm, 2006) (**evidence**). Phase 1 participants also noted that HCPs need to be taught the concept of SDM and the insulin PDA. Given that the researcher’s supervisors are both experts in the field of SDM and affiliated with the UMMC primary care clinic, hence there was expertise available to conduct the SDM training workshop **(clinic context).** In this strategy, a lecture would be given to HCPs focusing on the SDM concept to develop their knowledge and understand its benefits. In addition, they can understand that SDM and the insulin PDA use would lead to positive outcomes such as improved quality patient care and move away from the paternalistic approach **(mechanism: beliefs about consequences).** Conducting training workshop was also selected to address the barrier ‘HCPs being too busy to use the insulin PDA during consultation as there are too many patients’. There are studies that have shown that PDA use does not necessarily increase consultation time (Stacey et al., 2014). It might help save time in the future as informed decision making can be achieved quicker (Green et al., 2004) (**evidence**). One study suggested having a mentor or peer expert to demonstrate how the insulin PDA can be incorporated into a standard clinical consultation in order to allay fears over increased time (French et al., 2012). Participants from Phase 1 suggested that HCPs tailor PDA use to their patients, discussing only the information that the patient needs to know instead of going through the entire PDA **(mechanism: skills)** . HCPs can be informed of the information above during the training workshop to change their perception that PDA use would take more time than usual care. This strategy is intended to target doctors’ skills and their perception that they would not be able to use the insulin PDA within limited consultation times. |
| **Strategy: Involve patients’ family members or caretakers** | This strategy was selected to address the barrier ‘patient not being able to read or understand the insulin PDA’. Many participants from Phase 1 proposed this strategy based on their experiences when faced with patients who were illiterate during consultations. Doctors would be taught to ask patients if they have family members or friends to help them use the insulin PDA. The expected mechanism of action for this strategy is environmental context and resources **(mechanism).** |
| **Strategy: Framing/reframing** | This strategy was selected to help change HCPs’ perception that using the insulin PDA would take up a lot of time in an already a busy clinic setting. ‘Framing/reframing’ is defined as “*suggest the deliberate adoption of a perspective or new perspective on behavior (e.g.: its purpose) in order to change cognitions or emotions about performing the behavior”* (Michie et al., 2013, p. 20). A study found that one clinician viewed PDAs as a tool that could facilitate decision-making in on-going consultations that could help make durable decisions to "save time in the future" (Watson, Thomson & Murtagh, 2008) (**evidence**). In this strategy, the researcher would inform HCPs that using the insulin PDA might potentially help them reduce the consultation time needed to achieve an informed decision with patients. Eventhough the first consultation might take more time, insulin decision-making discussions over subsequent consultations may be shorter. Furthermore, when patients read the PDA at home before their next consultation, less time would be needed for these discussions. The expected mechanism of action for this strategy is beliefs about consequences **(mechanism).**  ‘Framing/reframing’ was also selected to change patients’ perception that using the insulin PDA meant they had to start using insulin. One participant from Phase 1 has highlighted that the insulin PDA should be introduced to patients in a positive and non-threatening manner. In this strategy, HCPs would be taught to emphasise to patients that using the insulin PDA did not mean that they had to initiate insulin but to help them make an informed decision about diabetes treatment. They would also be taught to inform patients that there are various diabetes treatment options and patients can read the insulin PDA at home and think about their decision. This was intended to make patients’ be more receptive towards using the insulin PDA. The expected mechanism of action for this strategy is beliefs about consequences **(mechanism).** |
| **Strategy: To engage patients in treatment discussions by getting them to ask questions and express concerns** | This strategy was selected to address the patient barriers namely ‘patient rely on doctor to make health decision’ and ‘patient are not confident to use the insulin PDA by themselves’. Literature has shown that strategies whereby patients were prepared for an active consultation with doctors or prepared an agenda for their clinic visit can improve patient engagement. For example, getting them to ask their HCPs three questions: 1) What are my options?, 2) What are the benefits and harms?, and 3) How likely are these? (Lloyd & Joseph-Williams, 2016; Tai-Seale, 2011) (**evidence**). When doctors engage with patients in discussions, patients may feel more supported and confident in their ability to use the insulin PDA. Thus, they will have more active participation in their health management. The expected mechanism of action for this strategy is reinforcement **(mechanism).** |
| **Strategy: Inform HCPs on the advantages of the insulin PDA use** | This strategy was selected to address the three barriers namely, ‘HCPs tend to make decision for their patients’, ‘HCPs wants to finish their work quickly’, and ‘HCPs are not motivated to try new innovations’. This strategy was also similar to the BCTs ‘Information about social and environmental consequences’ which was defined as “*Provide information (e.g: written, verbal, visual) about social and environmental consequences of performing the behavior”* (Michie et al., 2013, p. 8), and ‘Information about emotional consequences’, which was defined as “*Provide information (e.g: written, verbal, visual) about emotional consequences of performing the behavior”* (Michie et al., 2013, p. 9). A systematic review evidenced that PDA implementation is facilitated by HCPs’ perception that SDM and PDA will have a positive impact on the health care processes and patient outcomes (Gravel Legare & Graham, 2006) (**evidence**). Phase 1 participants highlighted the need to inform HCPs on the advantages of the insulin PDA use so that they would understand its purpose and be motivated to use it. This strategy aimed to inculcate the perception that insulin PDA use would result in positive social and emotional outcomes among doctors and nurses in order to gain their interest and motivation in practicing SDM and use the newly introduced insulin PDA. They may then move away from paternalistic approach and use the insulin PDA without trying to rush through their consultation in order to finish their work quickly. HCPs would be informed that using the insulin PDA could help improve their skills in exploring patient’s ideas, concerns and expectations about their health treatment, skills-providing information, and in discussing difficult decisions with patients thereby leading to improved patient-doctor communication. In addition, they might feel more satisfied with the quality of service given to patients and have an increased sense of accomplishment in their work. The expected mechanism of action for this strategy is beliefs about consequences **(mechanism).** |
| **Strategy: Juxtapose PDA in preferred language with patient’s PDA in their preferred language to help with translation** | This strategy was selected to teach HCPs on how to overcome the ‘language barrier’ when using the insulin PDA with patients. Phase 1 participants reported seeking help from ad-hoc interpreters like family members or other HCPs, to address language barriers during consultations. However, the researcher was concern that family members might misinterpret the information. Hence, it was decided to advise HCPs to juxtapose the PDA in their preferred language with a PDA in the patient’s preferred language to address the language barrier. One of the doctors in Phase 1 noted this strategy to be effective. This strategy would be less resource-intensive compared to hiring professional language interpreter service, which is not feasible based on UMMC primary care clinic’s tight operation budget as disclosed by participants in Phase 1 (**clinic context**). The expected mechanism of action for this strategy is environmental context and resources **(mechanism).** |
| **Strategy: Revise professional role** | This strategy has been defined as “*shift or revise roles among professionals who provide care, and, redesign job characteristics”* (Powell et al., 2015, p. 10) and was selected in this study to help address the barriers ‘HCPs are too busy as there are too many patients’, ‘Patients cannot read or understand the insulin PDA’, ‘Patients are not confident to use the insulin PDA by themselves’ and ‘HCPs will not use the PDA because they are not in-charge of the use of PDA in the clinic’. PDA implementation studies have shown that involving staff other than clinicians, such as nurses, in PDA implementation can help alleviate the time needed for doctors to use the PDA with patients (Joseph-Williams et al., 2017; Lin et al., 2013; Scalia, Elwyn & Durand, 2017) (**evidence**). At the UMMC primary care clinic, the diabetes educator is responsible for educating patients on insulin use, managing possible side effects such as hypoglyacemia and self-monitoring of blood glucose. Sometimes, they also received referrals from doctors to educate patients who were indecisive about starting insulin as they have the time, knowledge and skills to counsel patients. If they are trained, they can also execute the PDA use and SDM conversation with patients (**clinic context**). Participants from Phase 1 also suggested training diabetes educators to use the insulin PDA with patients to address doctors’ time constraints. Furthermore, diabetes educators could also be identify eligible patients for the insulin PDA and provide them with one for reading while they are waiting for their doctor’s consultation. This will enable effective use of patient’s waiting time and prepare them for insulin PDA discussions before seeing the doctor. Diabetes educators can also be assigned to monitor and order the supply of insulin PDA booklets given their specific role in terms of diabetes management in the clinic (**clinic context**). The expected mechanism of action for this strategy in this context is social/professional role and identity **(mechanism).** |
| **Strategy: Systematic documentation** | This strategy was selected to address the barrier ‘HCPs not able to see the same patient for follow up on the PDA’. Doctors at the UMMC primary care clinic have their own assigned patients but due to external postings, they might not be at the clinic at all times which makes it difficult to see the same patient. While getting doctors to use the insulin PDA with their assigned patients can ensure they see the same patient again, however, this would limit the reach of the PDAs to many patients. That is, a doctor might not give an eligible patient the insulin PDA as they are not the doctor’s assigned patient. In an academic healthcare setting, it is hard to have a system that can guarantee patients to see the same doctor every time. Until it is possible, the strategy to enhance information continuity is warranted where shared information links patient care between HCPs (Haggerty et al., 2003). Therefore, rather than focusing on ensuring doctors get to see the same patient, a strategy that focus on information continuity of care would be adopted. Phase 1 participants noted the need for systematic documentation of the insulin PDA use to ensure follow-up with patients and preventing doctors from giving the PDAs to patients who have received it before. Based on the clinic context, the UMMC primary care clinic has an EMR system which helps to facilitate patient information continuity whereby doctors in the clinic can see a patient’s medical history, relevant health information, and management by previous doctors **(clinic context).** The EMR system would be utilised to help facilitate the systematic documentation of the insulin PDA use. The expected mechanism of action for this strategy in this environmental context and resources **(mechanism).** |
| **Strategy: Provide feedback** | This strategy was selected to address the barrier ‘HCPs are not motivated to try new innovation’. Insulin PDA implementation studies show that HCPs are more motivated to use PDAs when they receive positive feedback from patients (Silvia & Sepucha, 2006), data on improvements of patient satisfaction, knowledge or other outcome measures (Arterburn, Westbrook, & Hsu, 2016; Feibelmann, Yang, Uzogara, & Sepucha, 2011; Hsu, Liss, Westbrook, & Arterburn, 2013; J. King & Moulton, 2013; Lloyd, Joseph-Williams, Edwards, Rix & Elwyn, 2013; Tietbohl et al., 2015) **(evidence).** Participants in Phase 1 also suggested providing feedback to HCPs to influence them to adopt the insulin PDA. This strategy aims to target social influences where high PDA adoption rates by their colleagues in the feedback may influence HCPs to do the same as they thought they are not doing as much like everyone else. The clinic’s monthly unit meetings also provide a suitable avenue for feedback provision given that most of the clinic staff would usually be present **(clinic context).** The expected mechanism of action for this strategy is social influences **(mechanism).** |
| **Strategy: Place the insulin PDA booklets in doctors’ consultation rooms** | This strategy was selected to address the barrier of ‘HCPs not knowing where to access the PDA’. One study showed that PDAs were adopted and distributed more when they were placed within HCPs’ reach (Lin et al., 2013) (**evidence**). Many Phase 1 participants suggested placing the insulin PDA booklets in the consultation rooms to enable doctors to access them easily. At the UMMC primary care clinic, staff nurses are responsible for replenishing medical supplies that are being used in the clinic hence this strategy could be included in the nurses’ work flow (**clinic context**). The expected mechanism of action for this strategy is environmental context and resources **(mechanism).** |
