# Supplementary material for: Developing an implementation intervention, and identifying strategies for integrating health innovations in routine practice: A case study of the implementation of an insulin patient decision aid
Source: PLoS One. 2024 Nov 15;19(11):e0310654. doi: 10.1371/journal.pone.0310654 (PMC11567623; doi:10.1371/journal.pone.0310654)
Supplement: S4 Table — (DOCX) [file pone.0310654.s004.docx]

**S4** **Table**

**Strategies operationalised to address the prioritised barriers based on the clinic context**

|  |  | **Dimensions to specifying a strategy** | | | | | |
| --- | --- | --- | --- | --- | --- | --- | --- |
| **Strategy** | **Target barrier** | **Actor** | **Action** | **Action target** | **Temporality** | **Dose** | **Implementation outcomes likely affected** |
| 1. Mandate change | I think HCPs will not use the PDA in the clinic because there is no clear directive from the top management to use the PDA | Head of department or clinic coordinator | Declare to all staff about clinic’s intention to implement and support the practice of SDM through the use of insulin PDA during unit meeting | Social influence: All clinic staff (doctors, diabetes educators, staff nurses, appointment clerks) would be influenced by the clinic authority to implement the insulin PDA | At the nearest unit meeting towards the insulin PDA training workshop | Two times per year following doctor turnover in clinic | - Reach (doctor) - Adoption (doctor) |
|  |  |  | Issue official letter to doctors and nurses to be involved in the insulin PDA implementation and to attend the training workshop |  | After the declaration of the insulin PDA implementation at the unit meeting and before the insulin PDA training workshop |  |  |
| 2. Training workshop | HCPs will not use the PDA in the clinic because they are too busy as there are too many patients | Researcher and supervisors | Teach doctors how they can use the insulin PDA within their limited consultation time  Inform doctor that they can discuss important information rather than going through the entire PDA with patient  Provide a 10-minutes reading session and quiz to familiarize HCPs with the insulin PDA | Doctors’ skills: Skills in using the insulin PDA during consultation  Doctors’ belief about capabilities: To improve doctors’ ability in using the insulin PDA within limited consultation time | After the official letter was issued to doctors and nurses | Two times per year following doctor turnover in clinic | - Adoption (doctor) |
|  | I think HCPs will not use the PDA in the clinic because they tend to make decisions for their patients instead of practising SDM | Researcher and supervisors | Provide a lecture on the SDM concept and what is insulin PDA | Doctors’ knowledge: Knowledge about SDM and the insulin PDA  Doctors’ belief about consequences:  Belief that SDM and the insulin PDA use would lead to positive outcomes such as quality patient care | After the official letter were issued to doctors and nurses | Two times per year following doctor turnover in clinic | - Adoption (doctor) |
|  | Patients will not use the PDA because they rely on doctors to make health decisions |  | Inform HCPs to encourage patients to be more involved in their health-decision making by getting them to ask questions and write down their concerns to discuss in their next visit |  |  |  | - Adoption (doctor) - Reach (patient) |
|  | Patients will not use the PDA because they are not confident to use the insulin PDA by themselves | Researcher and supervisors | Inform HCPs to encourage patients to be more involved in their health decision-making by getting them to ask questions and write down their concerns to discuss in their next visit | Doctors’ knowledge: Knowledge in using the insulin PDA with patients who are not confident in using it by themselves  Belief about capability:  Patients’ belief that they are capable of making decisions | After the official letter were issued to doctors and nurses | Two times per year following doctor turnover in clinic | - Adoption (doctor) - Reach (patient) |
| 3. Involve patients’ family members or caretakers (embedded within the training workshop) | Patients will not use the PDA because they cannot read or understand the insulin PDA | Researcher | Train doctors to  ask patients who are unable to read or understand the insulin DA if they have anyone (e.g. family members) who can help them | Doctor’s knowledge: Knowledge in using the insulin PDA | During the insulin PDA training workshop (Strategy: Conduct educational meeting) | Two times per year following doctor turnover in clinic | - Adoption (doctor) |
| 4. Framing/reframing (embedded within the training workshop) | HCPs are too busy to use the insulin PDA | Researcher | Inform HCPs that when patients use the PDA prior to consultation, less time will be needed to provide information to patients during consultation.  Inform HCPs that while the first consultation using the insulin PDA may take a longer time, insulin decision making over subsequent consultations may be shorter, and reduces delay in decision-making. | Doctors’ belief about consequences: Belief that insulin PDA would not increase but lead to a more effective use of their consultation time | During the insulin PDA training workshop (Strategy: Conduct educational meeting) | Two times per year following doctor turnover in clinic | - Adoption (doctor) |
|  | Patients will not use the PDA because they feel that the insulin PDA is a tool to persuade them to start insulin | Researcher and supervisors | Inform patients that the insulin PDA is not to persuade them to start insulin but to help them make informed decisions about diabetes treatment. | Doctors’ skills: Skills in delivering the insulin PDA to patient  Patients’ emotion: Patient emotion towards the insulin PDA | During the insulin PDA training workshop (Strategy: Conduct educational meeting) | Two times per year following doctor turnover in clinic | - Reach (patient) |
| 5. To engage patients in treatment discussions by getting them to ask questions and express concerns (embedded within the training workshop) | Patient rely on doctor to make health decision  Patient are not confident to use the insulin PDA by themselves | Doctors | Train doctors to engage patients in discussions about the insulin PDA. To encourage patients to ask questions and express concerns about treatment options | Doctor’s knowledge: Knowledge in using the insulin PDA | During the insulin PDA training workshop (Strategy: Conduct educational meeting) | Two times per year following doctor turnover in clinic | - Adoption (doctor) |
| 6. Inform HCPs on the advantages of the insulin PDA use (embedded within the training workshop) | HCPs tend to make decisions for their patients instead of practising shared decision making  HCPs want to finish their work quickly  HCPs are not motivated to try new innovations | Researcher | Inform HCPs of the advantages of practising SDM and insulin PDA use such as promoting patient centered care, improving patient-doctor communication, reducing delay in decision making, decrease consultation time, feeling more satisfied with quality of service given to patients and increased sense of accomplishment | Doctors’ and nurses’ knowledge about the advantages of using the insulin PDA | During the insulin PDA training workshop (Strategy: Conduct educational meeting) | Two times per year following doctor turnover in clinic | - Adoption (doctor) |
| 7. Juxtapose PDA in preferred language with patient’s PDA in their preferred language to help with translation (embedded within the training workshop) | Patients have difficulty to use the PDA with HCPs because of language barrier | Researcher | Inform HCPs they can use the PDA in their preferred language while patient are given the PDA in their own preferred language. During discussions, the patient can point out at areas that they want to discuss and the HCP can refer to their own insulin PDA version and clarify with patients | Doctors’ knowledge:  Doctor to be aware on how to use the insulin PDA when faced with language barrier | During the insulin PDA training workshop (Strategy: Conduct educational meeting) | Two times per year following doctor turnover in clinic | - Adoption (doctor) - Reach (patient) |
| 8. Revise professional roles | HCPs are too busy as there are too many patients  Patients cannot read or understand the insulin PDA  Patients are not confident to use the insulin PDA by themselves  HCPs will not use the PDA because they are not in-charge of the use of PDA in the clinic | Research and the  Head of department or clinic coordinator | Expand diabetes educators’ or staff nurses’ roles to engage patients in discussions about insulin initiation  Assign the diabetes educator as the person in-charge to identify patients who are eligible to use the insulin PDA, to give patients the insulin PDA ahead of consultation and keeping and monitoring the supplies of the insulin PDA booklets for the clinic | Diabetes educators’ or staff nurses’ social/professional role and identity: diabetes educators and staff nurses to embrace their role in conducting insulin decision-making counseling with patients using the insulin PDA | Prior to the implementation | One time | - Adoption (diabetes educator or staff nurses) |
| 9. Systematic documentation | HCPs are not be able to see the same patient to follow up on the PDA | Doctor | Make a note in patients’ medical notes in the EMR for PDA follow-up  Provide a follow-up appointment within 3 months to patients  Make a note in patient’s appointment card that PDA is given | Doctors’ memory, attention, decision processes: Doctors’ would be reminded or prompted to follow-up about the insulin PDA with patients | Throughout the implementation period | Every time an insulin PDA is given to a patient | - Adoption (doctor) |
|  |  | Appointment clerk | Make a note in the EMR (Remark section) that patient received PDA based on the note in the patient’s appointment card |  |  | Every time patient appointment card received indicate that insulin PDA has been given | - Adoption (doctor) |
| 10. Provide feedback | HCPs are not motivated to try new innovations | Researcher | Prepare individualised feedback report to all clinic staff | Social influences (social norm):  When a HCP saw high PDA adoption rates by their colleagues in the feedback, they may be influenced to do the same as they thought they are not doing as much like everyone else | Starting from the second month of the implementation period | Once per month (May to Oct: six times) until the end of the implementation period | - Adoption (doctors, diabetes educator, staff nurses) |
|  |  | Clinic coordinator | Provide feedback during unit meeting |  | Starting from the second month since insulin PDA use in the clinic |  |  |
| 11. Place the insulin PDA booklets in doctors’ consultation rooms | HCPs will not use the PDA in the clinic because they don't know where to get the PDA | Staff nurse | Place the insulin PDA booklets in doctors’ consultation rooms where they are within sight and easily reached by the doctors.  Replenish the insulin PDA supply in doctor's consultation room | Doctors environmental context and resources: Doctors’ access to the insulin PDA | Throughout the implementation period | One time. Ensuring the insulin PDA is available in all the consultation rooms.  Replenish whenever there is a lack of the insulin PDA booklets in the consultation rooms | - Adoption (doctor) |
