# Supplementary material for: Developing an implementation intervention, and identifying strategies for integrating health innovations in routine practice: A case study of the implementation of an insulin patient decision aid
Source: PLoS One. 2024 Nov 15;19(11):e0310654. doi: 10.1371/journal.pone.0310654 (PMC11567623; doi:10.1371/journal.pone.0310654)
Supplement: S1 Fig — (DOCX) [file pone.0310654.s005.docx]

**S5 Figure**

**The draft implementation intervention**

PRE-IMPLEMENTATION PERIOD

**Strategy: Mandate change**

**Strategy: Training workshop**

**Strategy: Provide feedback**

Diabetes educator to identify patient by screening patient medical record a day before patient appointment the next day

Doctor to select patient by screening their medical record during consultation

**Strategy: Systematic documentation**

Doctor makes a note in EMR and patient’s appointment card to indicate PDA has been given

If there is time, doctor goes through PDA in detail with patients

**Strategy: To engage patients in treatment discussions**

**Strategy: Juxtapose PDA in preferred language with patient’s PDA**

If there is no time, doctor to introduce PDA briefly to patients

Doctor to provide an appointment within 3 months for PDA follow-up

Doctor to refer patient to diabetes educator at the Diabetes education Counseling Unit for more explanation on PDA. Diabetes educator to go through insulin PDA with patients

Patient see appointment clerk to get next appointment date. Appointment clerk to make a note in EMR ‘Remark’ section to indicate patient received insulin PDA based on the note made by doctor in the patient appointment card

Patients bring PDA home to read

Patient return to clinic on decision or further discussion on PDA

Diabetes educator to approach identified patient in clinic

If there is time, diabetes educator can go through PDA with patients in diabetes room

If there is no time, diabetes educator to give PDA to patients for reading while waiting for consultation

Patient enter consultation room

IMPLEMENTATION PERIOD

**Strategy: Involve patients’ family members or caretakers**

**Strategy: Framing/reframing**

**Strategy: Inform HCPs of the advantages of using the insulin PDA**

**Strategy: Revise professional roles**

**Strategy: Place insulin PDA booklets in the doctors’ consultation rooms**
