# Supplementary material for: Developing an implementation intervention, and identifying strategies for integrating health innovations in routine practice: A case study of the implementation of an insulin patient decision aid
Source: PLoS One. 2024 Nov 15;19(11):e0310654. doi: 10.1371/journal.pone.0310654 (PMC11567623; doi:10.1371/journal.pone.0310654)
Supplement: Attachment — Submitted filename: Response to reviewers_25.2.2024.docx [file pone.0310654.s006.docx]

**Reviewer #1**

| **Reviewer comment** | **Response to reviewer** |
| --- | --- |
| 1. Could you provide more clarity on the specific evidence-based innovation being implemented in routine practice? | More details about the insulin PDA is now added into the manuscript:  Page 4  *In 2012 (with an update in 2014), an insulin PDA booklet was developed in Malaysia to promote patient centered care through fostering SDM practices and to help patients to make informed decisions about diabetes treatment [25, 26]. The insulin PDA was developed based on evidence from a systematic review, decision support theories and frameworks, and the needs of local healthcare policymakers, clinicians, and patients [25]. The booklet has 12 pages divided into five sections. The first and second sections provide information about diabetes and insulin, and the pros and cons of available treatment options. The third section entails the values clarification exercise where patients share what is important to them about insulin initiation and their concerns with insulin. Subsequently, patients are asked explicitly if they needed more support (section four) before finally making a decision (section five). The insulin PDA is available in the Malay, Chinese, Tamil, and English languages to cater to the Malaysian multiethnic and multilingual population. According to the International Patient Decision Aids Standards Collaboration, which assesses the quality and standards of a PDA, the insulin PDA scored 7/7 for decision aid criteria and 5/8 for the risk of bias criteria [27].* |
| 2. Can you elaborate on why the Action Cycle in the Knowledge to Action framework was chosen as the guiding framework for intervention development? | The Action Cycle outlines steps that include assessment of barriers to knowledge followed by selecting, tailoring and implementing intervention. A Cochrane systematic review found that implementation is more likely to be effective in changing professional practice and healthcare outcomes if the strategies are tailored based on identified barriers and facilitators [1]. Hence, the Action Cycle was chosen as the guiding framework for intervention development.  We have now included this explanation in the text:  Page 4  *The development of the insulin PDA implementation intervention was divided into two phases incorporating specific steps in the Action Cycle of the Knowledge to Action framework. Phase 1 was assessment of barriers to the insulin PDA implementation. This phase corresponds to step 3 of the Action Cycle ‘Assess barriers to knowledge use’. Phase 2 corresponds to step 4 in the Action Cycle ‘Select, tailor, implement intervention’. This phase involved selection and tailoring of strategies to overcome the barriers identified in phase 1. These two steps within the Action Cycle were used for the implementation intervention development as implementation is more likely to be effective in changing professional practice and healthcare outcomes if the strategies are tailored based on identified barriers and facilitators [31].* |
| 3. How were participants recruited for the qualitative interviews, and what were the inclusion criteria? | Information about the participant inclusion criteria and recruitments processes are now included. References of prior publications are now included for readers to refer to the detailed information of the participant recruitment process.  Page 5  Healthcare policymakers, HCPs, and patients who fulfilled the study inclusion criteria were invited to participate in the study. The inclusion criteria for healthcare policymakers were those who were responsible for or involved in making decisions on whether a particular health intervention should be implemented in their healthcare settings. They set standards of care and implement programmes to improve diabetes care. These included the director-level hospital manager, endocrine and primary care medicine policymakers, hospital matrons, and clinic managers. HCPs included were doctors, diabetes educators and staff nurses who were involved in advising patients about insulin initiation. Patients with type 2 diabetes who have been advised to start insulin or were currently using insulin and those who had been seeking diabetes treatment in the clinic for more than one year were recruited. In order to achieve maximum variation, we recruited participants from different socio-demographic backgrounds (ethnicity, gender) and included those with and without experience in using the PDA. Healthcare policymakers and HCPs who fulfilled the study inclusion criteria were invited to participate through telephone calls. Doctors in the clinics helped identify eligible patients and referred them to the researcher. Interview appointments were then made with eligible participants. Detailed information about the participant recruitment process have been reported in prior publications [35-37]. |
| 4. Can you provide more detail on the interview guide developed based on the Theoretical Domains Framework? | More details are now provided on the interview guide. References of prior publications are now included for readers to refer to the detailed summary of interview guides for both healthcare policymaker and HCPs, and, patients.  Page 6  The interviews were conducted with the aid of a semi-structured interview guide which was developed based on the TDF [34], literature review on factors influencing PDA implementation, and discussions among researchers. The interview guide comprised of five sections, and they were: the innovation - insulin PDA, individuals, inner context – the clinic, and the outer context – the healthcare system. Questions and prompts were created according to the domains in the TDF within each of the sections. For example, under the Individual section, the question ‘Do you think that insulin PDA will affect your/HCP’s role? How?’ was developed to address the social professional role and identity domain in the TDF. Under the inner context – clinic section, the question ‘How do you think your organisation’s working culture will affect the implementation of the PDA?’ was developed to address the environmental context and resources domain in the TDF. The summary of the interview guide for policymakers and HCPs, and patients can be found in prior publications [35, 36]. The interview guides were adapted according to the participants’ roles: healthcare policymakers, HCPs, and patients with type 2 diabetes. |
| 5. How were the barriers and facilitators categorized into HCP, patient, organizational, and innovation factors? | The barriers and facilitators were categorised into HCP, patient, organizational, and innovation factors through thematic analysis. Information about the how the barriers and facilitators categorized into HCP, patient, organizational, and innovation factors is now included.  Page 6  Interviews were audio-recorded and transcribed verbatim. Thematic analysis was conducted using the NVivo qualitative analysis software. **During the data analysis, the interview transcripts were read in detail and then coded. A code is short text that represents the meaning of a text segment. Codes that have similar meaning were grouped together to form a category, and later the categories were compared and merged into bigger themes: HCP, patient, organizational, and innovation.** |
| 6. Could you explain the process of multivoting in more detail, including how priorities were determined and by whom? | Detailed information of the multivoting process is now included.  Page 7  **The multivoting process involved the clinic stakeholders (n=12) from the UMMC primary care clinic; the clinic coordinator, three doctors, two diabetes educators, three staff nurses and three patients in a two-round voting process, which took approximately three hours. During the multivoting exercise, the participants were informed that the purpose of the exercise was to narrow down the list of barriers to the insulin PDA implementation, that were identified in a previous qualitative study (Phase 1) so that most important barriers can be identified for development of targeted intervention. The researcher also presented the insulin PDA and the concept of SDM to the participants using PowerPoint slides. In addition, examples of how the insulin PDA can be used in the clinic were also provided to enable the participants to think about the processes of the insulin PDA implementation, and what are the main barriers that could hamper its implementation in the UMMC primary care clinic. Once the participants were familiar with SDM and the insulin PDA, a Voting Form was distributed which consisted of the barriers that were identified from Phase 1 (S1 Appendix).** Facilitators identified in phase 1 were revised as barriers by expressing the idea in the form of negative statement. For example, ‘Directive from higher authority’ was revised to ‘There is no clear directive from higher authority to use the insulin PDA’. This allowed the researcher to find out if the ‘opposite’ or absence of the facilitator would be a prioritised barrier for implementation.  **The multivoting process is illustrated in Figure XX. Participants were given individual voting forms and asked to vote privately to prevent them from influencing one another. Once voting forms were completed, they were immediately entered into an excel spreadsheet and tabulated. The first round of voting resulted in 18 barriers remaining in the list. Then, participants were asked to vote for their top 10 highest priority items of the list. This reduced the number of barriers to approximately 10 items, which the researchers discussed and decided would be feasible for the development of the tailored intervention prior to the multivoting process.**  **Step 1: Round 1 voting**   - Using the Voting Form developed (49 barriers), the participants voted as many items as they wish.   **Step 2: Round 1 analysis**   - Once votes are tabulated, barriers with a vote count equivalent to or more than half the number of participants remain on the list. - Second Voting Form generated.   **Step 3: Round 2 voting**   - Using the second Voting Form, (18 barriers) the participants voted for their top 10 highest priority items of the list   **Step 4: Round 2 analysis**   - Round 1 analysis repeated. - Key barriers identified.   **Figure 2: The multivoting process** |
| 7. Can you provide examples or case studies illustrating how each of the identified strategies can address the prioritized barriers in the implementation of the insulin PDA? | This information is now included in the manuscript and Appendix. The strategies were chosen based on evidence in the literature and the feasibility to implement them in the clinic context. Page 18 Similar strategies were consolidated. Finally, a total of 11 strategies were selected by the researchers based on evidence in the literature, the feasibility to implement in the clinic context, and their expected mechanism of action. The strategies are listed in Table 3. Table 3: Implementation strategy selected and their expected mechanisms of action **Strategy** \| **Expected mechanism of action**; 1. Mandate change \| Social influence; 2. Training workshop \| Knowledge, awareness, skills, beliefs about consequences; 3. Involve patients’ family members or caretakers \| Environmental context and resources; 4. Framing/reframing \| Beliefs about consequences; 5. To engage patients in treatment discussions by getting them to ask questions and express concerns \| Reinforcement; 6. Inform HCPs on the advantages of the insulin PDA use \| Beliefs about consequences; 7. Juxtapose PDA in preferred language with patient’s PDA in their preferred language to help with translation \| Environmental context and resources; 8. Revise professional roles \| Social/professional role and identity; 9. Systematic documentation \| Environmental context and resources; 10. Provide feedback \| Social influences; 11. Place the insulin PDA booklets in doctors’ consultation rooms \| Environmental context and resources *For example, the strategy mandate change was chosen to address the barrier not having a clear directive from the top management to use the insulin PDA. This strategy was selected because it has been shown that when organisational leader showed clinicians that SDM was an important organisational priority, it led clinicians to believe that SDM was part of the organisation’s work practice and not something that was imposed on them [2-4] (****evidence****). Based on the clinic context, the Head of Department or the clinic coordinator organise monthly unit meetings, where all clinic staff (doctors, diabetes educators, staff nurses, appointment clerks) gathers to discuss matters related to the clinic, Hence, this provides opportunity to implement this strategy in a unit meeting (****clinic context****). Participants from Phase 1 also noted that directive from higher authority was an effective way to implement the insulin PDA. Furthermore, this strategy was perceived to be more feasible compared to other strategies such as incorporating the insulin PDA use as part of key performance index or standard operating procedure. This is because the latter strategies would require approvals from hospital authorities and bureaucratic paperwork that are time consuming (****clinic context****). Declaration from the clinic authority may influence the clinic staff to see that the insulin PDA implementation is a clinic’s priority and will influence them to be involved in the implementation. The expected mechanism of action for this strategy is social influence (****mechanism of action****). The justification for all the strategies selected, their expected mechanisms of action, and how they can address the barriers can be found in S3 Appendix.* **Response to reviewer:** It should be noted that not all strategies have evidence that they would be effective as some strategies were developed based on the needs for this study such as Involve patients’ family members or caretakers, Juxtapose PDA in preferred language with patient’s PDA in their preferred language to help with translation, and Systematic documentation. |
| 8. How were the selected strategies operationalized and integrated into the patient care pathway, and were there any challenges encountered during this process? | More information is now provided on how the selected strategies were operationalised. No challenges were encountered during this process.  Page 8  *Subsequently, each of the discrete strategy that was selected was operationalised. The reason for specifying the strategies was to allow the researcher to determine if the strategies would be feasible to be carried out in the clinic context. Also, when a strategy is described in sufficient detail, it allows for scaling or replication in other research and practice [42]. The operationalisation was based on recommendations by Proctor et al., (2013) who suggested that implementation strategies should be specified according to seven dimensions: the actor, the action, the action target, temporality, dose, the implementation outcome affected and, the justification for selecting the strategy [42]. However, in this study, justification for selection of the strategy had already been described in step 2c, thus, was not included as part of the operationalisation.* ***The operationalisation of the strategies was done by the researchers who took into account the clinic’s context and resources. One of the researchers (CJN) is a family medicine specialist practising in the clinic and was able to provide input on the workflow processes in the UMMC primary are clinic.*** *Once the implementation strategies were operationalised, they were embedded into the existing diabetes patient care pathway in the clinic.* |
| 9. What feedback was received during the clinic stakeholders meeting, and how was the final implementation intervention refined based on this input? | We have provided this information in the manuscript as follows:  Page 20  One prominent issue that was raised by the participants on the draft intervention was that diabetes educators would no longer be available in the clinic as they would be transferred to another unit. Hence, the strategies and processes that would involve diabetes educators to execute the pre-visit insulin PDA use, and referral of patients to diabetes educators for detail use of the insulin PDA would not be feasible. It was decided that the strategy ‘Define roles and responsibilities’ would replace the strategy ‘Revise professional roles’ where all the clinic staff (doctor, staff nurses, and appointment clerks) would be informed of their specific task in the insulin PDA implementation. In this strategy, all the clinic staff (doctor, staff nurses, and appointment clerks) would be informed of their specific task in the insulin PDA implementation (action). This strategy aimed to make HCPs embrace the implementation tasks given to them as part of their professional roles (action target). Clear role definitions have been reported to facilitate PDA implementation [22, 67] (evidence) by enabling clinic staff to be aware of one’s own responsibility but at the same time also aware of other’s unique contribution to the implementation and work together to accomplish tasks through shared responsibility [67]. Doctors and nurses would be informed explicitly of their specific implementation tasks during the training workshop. The flow of the insulin PDA implementation will be explained so that everyone would be aware of their responsibilities. Apart from doctors and nurses, appointment clerks would also be informed of their administrative role of to indicate that the insulin PDA had been given to patients in the patient medical record. The assignment of implementation tasks to various HCPs would be done one time (dose) prior to the implementation (temporality). This strategy aimed to make HCPs embrace the implementation tasks given to them as part of their professional roles. The expected mechanism of action for this strategy is social/professional role and identity.  Table 5 shows the finalised strategies selected to overcome specific barriers, while Figure 3 shows the finalised implementation intervention. |
| 10. Can you discuss potential sustainability and scalability considerations for the implementation intervention beyond the initial phase? | We have now included discussions on the potential sustainability and scalability considerations for the implementation intervention  Page 25  *The implementation intervention development process in this study considered future sustainability and scalability. During strategy selection and operationalisation, while having a champion had been found to be an effective strategy for PDA implementation [5], this strategy was not selected for this study because the UMMC primary care clinic has a regular turnover of medical officers due to it being part of an academic hospital. Hence, a trained champion might leave the clinic which rendered this strategy unsustainable. In addition, the researchers also ensured that the right staff were chosen to carry out the strategies as well as the timing and dosage of strategies. For example, the strategy ‘educational workshop’ would be conducted twice a year (dose) following doctor turnover in the clinic to ensure new doctors are trained on using the insulin PDA. Furthermore, engagement of stakeholders throughout various steps in this study in ensuring that strategies that were chosen target the right barriers, that they were feasible to be implemented in the clinic context, and integrating the implementation process with the diabetes patient care pathway in the clinic enhances the sustainability and scalability of the implementation intervention.* |
| 11. What are the anticipated mechanisms through which each strategy is expected to facilitate the implementation of the insulin PDA? | We have now provided information on the anticipated mechanisms of action for each of the strategies: Page 18 *Finally, a total of 11 strategies were selected by the researchers based on evidence in the literature, the feasibility to implement in the clinic context, and their expected mechanism of action. The strategies are listed in Table 3.* *Table 3: Implementation strategy selected and their expected mechanisms of action* **Strategy** \| **Expected mechanism of action**; 12. Mandate change \| Social influence; 13. Training workshop \| Knowledge, awareness, skills, beliefs about consequences; 14. Involve patients’ family members or caretakers \| Environmental context and resources; 15. Framing/reframing \| Beliefs about consequences; 16. To engage patients in treatment discussions by getting them to ask questions and express concerns \| Reinforcement; 17. Inform HCPs on the advantages of the insulin PDA use \| Beliefs about consequences; 18. Juxtapose PDA in preferred language with patient’s PDA in their preferred language to help with translation \| Environmental context and resources; 19. Revise professional roles \| Social/professional role and identity; 20. Systematic documentation \| Environmental context and resources; 21. Provide feedback \| Social influences; 22. Place the insulin PDA booklets in doctors’ consultation rooms \| Environmental context and resources |
| 12. Can you discuss the potential implications of the study findings for policy and practice, including recommendations for healthcare organizations and policymakers? | The potential implications of the study findings for policy and practice are now included.  Page 26  **Potential implications of study findings for policy and practice**  *This study highlights the potential strategies that can be implemented to integrate the use of insulin PDA in routine clinical practice. Healthcare providers would require training and support to integrate the insulin PDA in their practice. Effective implementation of the insulin PDA through the strategies identified can facilitate doctors in discussing diabetes management with patients thereby improving patient-doctor communication. Policymakers need to address systemic barriers through targeted policy changes, infrastructure improvements, or resource allocations for effective implementation of decision support tools. The use of PDA needs to be included in healthcare policies to support shared decision making practices among healthcare providers. In addition, policies can also be adjusted to consider patient satisfaction and engagement through the use of PDA as important metrics when evaluating the success of provision of quality health care.* |
| 13. How do you plan to evaluate the effectiveness and impact of the implementation intervention in real-world settings? | The evaluation of the effectiveness and impact of the implementation intervention has been conducted and the manuscript is currently under revision for publication in another journal. |
| 14. Could you provide more information on the demographics and characteristics of the participants in the qualitative interviews? | More information on the demographics and characteristics of the participants in the qualitative interviews is now provided:  Page 9  *Among the healthcare policymakers and the HCPs, their mean age was 39.4 (± standard deviation (SD) 9.7) years (range: 28-57) and 86.8% were female. The mean duration of practice since entering workforce was 14.7 (± SD 10) years (range: 3-35). Six of them had experience using the insulin PDA from the acceptability study. Among the 31 patients, their mean age was 63.0 (± SD 11.6) years (range: 36-79) and there was slightly more male participants (51.6%). The mean duration of having diabetes was 12.8 (± SD 7.9) years (range: 4 months – 30 years) while the mean duration of them seeking diabetes treatment in the clinic was 8.58 (± SD 5.9) years (range: 2 months – 20 years). Almost half of them were Malays (48.4%) followed by Chinese (32.3%) and Indians (19.3%). Slightly more than half of the patients (54.8%, n=17/31) were using insulin at the time of the interview.* |
| 15. Are there any biases or limitations associated with the qualitative data collection process, and if so, how were they addressed? | We have now added a few more limitations to the qualitative data collection process and the measures that were taken to offset them.  Page XX  ***This study has a few limitations. In Phase 1, some participants may not know the concept of SDM and PDAs. To address this barrier, participants were shown a video demonstrating how the insulin PDA can be used in a consultation, which may have biased their views about how to implement the PDA. Secondly, social desirability bias may have been present with participants who wanted to support the use of the PDAs or those that wanted to provide a favourable view of the clinic. This was addressed by ensuring participants that the data would be kept confidential and their participation would not affect their work, career or health care provision (for patients).***  ***In Phase 2,*** *the development of implementation intervention in this study involved only a few patient stakeholders and this may have affected the representativeness of the findings; i.e., when HCPs outnumber patients, it renders clinicians more visibility and perhaps they are also more vocal in the design of the intervention [6]. Nevertheless, care was taken to ensure that every stakeholder had a chance to voice their opinions by posing questions related to their roles in the implementation. This study also did not involve clinic staff such as medical assistants, and appointment clerks as part of the intervention development process given their limited roles in the implementation.* |

**Reviewer #2**

| How were the 11 strategies identified to address the prioritized barriers, and what criteria were used for their selection? | The 11 strategies were identified through three steps: Step 2a) searching for potential strategies, 2b) understanding the clinic context, and 2c) mapping strategies to prioritised barriers. The criteria for selection were effectiveness and feasibility.  This information has been provided in the following texts:  Page 8  *Using all the information gathered from the literature and understanding of the clinic context, the researchers then decided on the potential strategies to be utilised to overcome the prioritised barriers. The researcher went through each of the strategies identified and assessed its appropriateness* ***based on evidence of its effectiveness and feasibility in implementing in the UMMC primary care clinic.***  Page 8  *A clinic stakeholders meeting was conducted to discuss and finalise the implementation intervention. Clinic stakeholders who participated in the multivoting exercise in Step 1 were invited. The participants were engaged in an open discussion to discuss and come to a consensus on the implementation intervention drafted by the researchers. The proposed strategies to overcome the prioritised barriers, and the PDA implementation flow diagram were presented and explained in detail. Then,* ***participants were asked to take their time to deliberate if the draft intervention would be effective and feasible to be implemented in the clinic setting.*** *Every stakeholder (doctors, diabetes educators and staff nurses) was also asked to peruse their implementation tasks.* |  |
| --- | --- | --- |
| Can you discuss any potential unintended consequences or limitations associated with the implementation of the identified strategies? | We have now included this information in the manuscript.  Page 25  *There is a possibility that some of the strategies identified may have unintended consequences. This study adopted the strategy ‘Provide feedback’ to motivate HCPs to adopt the insulin PDA. The expected mechanism of action for this strategy is social influence where doctors would be motivated when they know their colleagues are also using the insulin PDA. However, there is a possibility that doctors may feel pressured when receiving feedback on their performance and get stressed if they have a poorer performance when compared to other colleagues.*  *Another strategy that may cause unintended consequences is “Place the insulin PDA booklets in doctors’ consultation rooms”. For patients who are already averse to insulin therapy, the sight of the insulin PDA may make them feel uncomfortable or forced to start insulin therapy.* | |
| Were there any notable differences in the barriers and facilitators identified among different stakeholder groups (e.g., healthcare policymakers, doctors, patients)? | We did not explicitly make a comparison of findings between the different stakeholder groups as our aim was to explore all the potential barriers to the insulin PDA implementation and then conduct the multivoting process to prioritise the more important barriers for development of the implementation intervention. | |
| Can you provide more information on the training workshop strategy, including its content, duration, and intended audience? | Given the length of the manuscript, we could not include the minute detailed information on the training workshop strategy as well as other strategies in the main manuscript. However, the information is provided in S4 Appendix: Strategies operationalised to address the prioritised barriers based on the clinic context. | |
| How were the roles and responsibilities defined for the various stakeholders involved in the implementation of the insulin PDA? | Information on how the roles and responsibilities were defined for the various stakeholders is now provided  Page 20  In this strategy, all the clinic staff (doctor, staff nurses, and appointment clerks) would be informed of their specific task in the insulin PDA implementation (**action**). This strategy aimed to make HCPs embrace the implementation tasks given to them as part of their professional roles (**action target**). Clear role definitions have been reported to facilitate PDA implementation [22, 67] (**evidence**) by enabling clinic staff to be aware of one’s own responsibility but at the same time also aware of other’s unique contribution to the implementation and work together to accomplish tasks through shared responsibility [67]. Doctors and nurses would be informed explicitly of their specific implementation tasks during the training workshop. The flow of the insulin PDA implementation will be explained so that everyone would be aware of their responsibilities. Apart from doctors and nurses, appointment clerks would also be informed of their administrative role of to indicate that the insulin PDA had been given to patients in the patient medical record. The assignment of implementation tasks to various HCPs would be done one time (**dose**) prior to the implementation (**temporality**). This strategy aimed to make HCPs embrace the implementation tasks given to them as part of their professional roles. The expected mechanism of action for this strategy is social/professional role and identity. | |
| Can you elaborate on the process of placing the insulin PDA in the consultation room and its potential impact on patient-provider interactions? | Given the length of the manuscript, we could not include detailed information on the strategies. However, the information on the process of placing the insulin PDA in the consultation room is provided in S4 Appendix: Strategies operationalised to address the prioritised barriers based on the clinic context. S4 Appendix: Strategies operationalised to address the prioritised barriers based on the clinic context. \| \| **Dimensions to specifying a strategy** \| \| \| \| \|; **Strategy** \| **Target barrier** \| **Actor** \| **Action** \| **Action target** \| **Temporality** \| **Dose** \| **Implementation outcomes likely affected**; 11. Place the insulin PDA booklets in doctors’ consultation rooms \| HCPs will not use the PDA in the clinic because they don't know where to get the PDA \| Staff nurse \| Place the insulin PDA booklets in doctors’ consultation rooms where they are within sight and easily reached by the doctors. Replenish the insulin PDA supply in doctor's consultation room \| Doctors environmental context and resources: Doctors’ access to the insulin PDA \| Throughout the implementation period \| One time. Ensuring the insulin PDA is available in all the consultation rooms. Replenish whenever there is a lack of the insulin PDA booklets in the consultation rooms \| - Adoption (doctor) **Response to reviewer:** The potential impact of the placement of the insulin PDA in the consultation room in patient-provider interactions is now provided: Page 25 Another strategy that may cause unintended consequences is “Place the insulin PDA booklets in doctors’ consultation rooms”. For patients who are already averse to insulin therapy, the sight of the insulin PDA may make them feel uncomfortable or forced to start insulin therapy. | |
| Were there any specific challenges or considerations related to engaging patients in treatment discussions during the implementation process? | This paper reports on the processes on the development of the implementation intervention. The intervention has yet to be implemented in this manuscript. | |
| Can you discuss how the identified strategies align with existing evidence on implementation science and healthcare quality improvement methodologies? | Information on how the identified strategies align with existing evidence on implementation science and healthcare quality improvement methodologies is now included in the Discussion section:  Page 25-26  Many strategies identified in this study align with implementation science and healthcare quality improvement methodologies. For example, implementation science and healthcare quality improvement emphasizes on the importance of continuous monitoring and feedback for effective implementation. The strategy to provide feedback identified in this study helps to provide ongoing assessment of the implementation process for doctors, and ensuring that they are aware of their adoption and fidelity to the implementation protocol. Having explicit mandates is also a core principle of implementation science and quality improvement methods to drive the adoption of evidence-based practice. Leadership support can help to facilitate change, overcoming resistance, and creating a supportive environment for new practices. The strategy mandate change in this study aims to influence clinic staff to believe that the insulin PDA implementation is a clinic priority hence they would want to be involved in the implementation. | |
