# Supplementary material for: Developing an implementation intervention, and identifying strategies for integrating health innovations in routine practice: A case study of the implementation of an insulin patient decision aid
Source: PLoS One. 2024 Nov 15;19(11):e0310654. doi: 10.1371/journal.pone.0310654 (PMC11567623; doi:10.1371/journal.pone.0310654)
Supplement: Attachment — Submitted filename: Response to reviewer 2.docx [file pone.0310654.s007.docx]

| **No** | **Reviewers’ comment** | **Response to reviewer** |
| --- | --- | --- |
| 1. | Since PDA is our implementation intervention, it is the main point to be considered. As mentioned about the PDA and its booklet, various reviews have been cited regarding development and application in page 4, its cost-effectiveness and clinical efficacy were not mentioned elsewhere. I suggest reviewing some literature and highlighting. | The cost effectiveness and clinical efficacy **specifically for the insulin PDA** has not been empirical studied. However, we believe that in general, the insulin PDA is effective given the established evidence on the efficacy of 105 PDAs in a Cochrane systematic review (Stacey et al., 2017). However, to date the cost-effectiveness of PDAs from patient, organisation, and society perspective is still unclear as shown in a systematic review (Scalia et al., 2020).  We how now included in the information above in the Introduction section:  Page 1  PDA use can increase patient involvement in decision-making, reduce decisional conflict, and has a positive effect on patient-doctor communication [6], although its cost effectiveness from patient, organisation, and society perspective is still unclear [7]. |
| 2. | It would be better to develop a figure of theory of change, that may reflect and synthesize all the steps, including formative and implementation phases, major barriers and strategies developed, and findings/conclusion. This may help readers understand easily. | We have removed Figure 1: The implementation intervention development steps of this study, and now provide the study theory of change that included the relevant steps in the development of the implementation intervention  Page 5  Figure 1: Study theory of change and the implementation intervention development steps of this study |
| 3. | Since this is implementation research, It would be better to reflect this in the title, as also, our one of the reviewers has suggested. | We have now revised the title of the study as following:  *Developing an implementation intervention, and identifying strategies for integrating health innovations in routine practice: a case study of the implementation of an insulin patient decision aid* |
| 4. | The title of manuscript may require minor revision as the study mainly focused on identifying implementation strategies and developing Implementation Intervention as an innovative approach rather than having use of innovation and integrating it into routine practice. | We have now revised the title of the study as following:  *Developing an implementation intervention, and identifying strategies for integrating health innovations in routine practice: a case study of the implementation of an insulin patient decision aid* |
| 5. | The study included urban government teaching hospital and community clinics, as different setting there might be different facilitators and barriers, thus how your study justifies the outcome ‘intervention’ for different settings as a whole? | In Phase 1, we explored the barriers to the insulin PDA implementation for both the urban government teaching hospital and community clinic to get a comprehensive understanding on the potential barriers to the insulin PDA implementation. The barriers that were identified were used for development of a Voting form (S1 Appendix 10). This voting form can be utilized by any setting that may want to implement the insulin PDA, and through the multivoting process, the most important barriers to implementation for the specific setting can be identified. In this study, we aimed to conduct a pilot implementation of the insulin PDA at the UMMC primary care clinic before scaling up to other settings. While the outcome ‘intervention’ would be more relevant to the UMMC primary care clinic, we believe that the it can also be applied to other setting such as the community clinics. |
| 6. | ‘Understanding clinic context’ seems very pivotal step/phase in this study, thus what major areas were considered while examining clinic context that supports for further generalization of study? | For ‘understanding clinic context’, the major areas that were investigated during the observations and via informal conversation with the clinic staff were to understand the type 2 diabetes patient **care pathway (**e.g., how were patients offered insulin therapy in terms of where, by who, when; what happens to patients when a decision about insulin is made?), and the **availability of resources** (e.g., what were the resources available in the clinics such as infrastructure and staff); what were the specific roles played by the various healthcare providers in the clinic). Knowing this information allowed the researcher to select strategies that would be appropriate to implement in the clinic, including incorporating them into the clinic’s daily work processes without requiring too many changes.  We now included this information in the manuscript:  Page 8  Step 2b: Understanding clinic context  There was a need to understand implementation context to know which strategy would be feasible to be implemented, hence, WTT conducted observations and engaged in informal conversation with the clinic staff to understand the type 2 diabetes patient care pathway, and the availability of resources. The information gathered enabled the selection of strategies that would be appropriate and feasible to implement in the clinic, including incorporating them into the clinic’s daily work processes without requiring too many changes. The key clinical contexts gathered for informing the insulin PDA implementation were:   - How were patients offered insulin therapy? (e.g.: where, by who, when) - What happens to patients when a decision about insulin is made? - What were the specific roles played by the various HCPs in the clinic? - What were the available resources in the clinics (i.e.: infrastructure, staff)? |
